# Machine learning-based analysis identifies glucose metabolism-related genes ADPGK as potential diagnostic biomarkers for clear cell renal cell carcinoma

**DOI:** 10.3389/fonc.2025.1559887

**Published:** 2025-09-16

**Authors:** Tie Li, Shijin Wang, Guandu Li, Xiaochen Qi, Guangzhen Wu, Xiangyu Che

**Affiliations:** Department of Urology, The First Affiliated Hospital of Dalian Medical University, Dalian, China

**Keywords:** glucose metabolism, clear cell renal cell carcinoma (ccRCC), machine learning, ADP-dependent glucokinase (ADPGK), immune infiltration

## Abstract

**Introduction:**

Clear cell renal cell carcinoma, with its high morbidity and mortality, is one of the more difficult diseases in the world and still lacks an effective therapeutic target. The primary way they break down glucose is through aerobic glycolysis, which leads to energy acquisition and synthesis of the material base required for cell growth. Although targeting glucose metabolism has driven the development of a variety of tumour therapies, the specific regulatory mechanisms remain unclear. Therefore, based on machine learning analysis algorithms, we analysed the correlation between glycometabolic pathways and ccRCC in the REACTOME database and verified the impact of the key gene *ADPGK* on the prognosis of ccRCC.

**Methods:**

We utilised a total of 89 gene collections of glucose metabolism pathways from the REACTOME (https://reactome.org/) database as the data base for our study. To uncover potential therapeutic target genes, we adopt three machine learning algorithms (LASSO, RF, and Boruta). We reassigned the 7 screened genes based on gene expression differences between cancer and paracancerous tissues, and applied an unsupervised consensus clustering algorithm to establish a typology based on the expression of glucose metabolism-related genes (*ADPGK*). We then validated the link between *ADPGK* and cancer cell invasion and metastasis by *in vitro* experiments on ccRCC cell lines.

**Results:**

We identified *ADPGK* as a key gene for the glucose metabolism pathway and suggested that it may promote invasion and metastasis of ccRCC. In addition, based on the results of immune infiltration, *ADPGK* was observed to significantly affect the immune response in ccRCC. Our results suggest that the implementation of therapeutic strategies based on key genes of glucose metabolism may bring new hope for ccRCC patients.

**Discussion:**

Our results suggest that targeting the glucose metabolism pathway can kill ccRCC cells. *ADPGK*, a gene related to glucose metabolism, may be an important biomarker for the diagnosis and characterization of ccRCC. However, whether *ADPGK* affects glycolysis in ccRCC, and the mechanism by which glycolysis is regulated is not clear. This is the direction of further research in the future.

## 1 Introduction

Renal cell carcinoma (RCC) is one of the common urological malignancies in adults, accounting for 2-3% of all adult malignancies, with clear cell renal cell carcinoma (ccRCC) accounting for approximately 80% of RCC cases (1). Although significant progress has been made in the systemic treatment of this group of tumours, multiple challenges remain to reduce mortality, such as the lack of clinically available biomarkers and insufficient understanding of the molecular mechanisms of ccRCC (2, 3). Therefore, in-depth exploration of potential therapeutic targets and biomarkers for ccRCC is crucial, which will help improve patient survival.

Studies have shown that glucose metabolism is strongly associated to tumour development, migration and drug tolerance, therefore, intervention regimens targeting tumour glucose metabolism have become the focus of exploration (4). Even under aerobic conditions, tumour cells are able to produce adenosine triphosphate (ATP) via glycolysis. In the 1920s, Otto Warburg was the first to notice this sight, which has since been named the “Warburg effect” (5). The Warburg effect describes the shift in energy provision from oxidative phosphorylation to glycolysis and the production of large amounts of pyruvate and lactate by tumour cells under aerobic conditions (6). RCC, particularly ccRCC, is often considered a metabolic disease that is prominently marked by alterations in key genetic loci in the metabolic pathway (7). These mutations play a role in the regulation of processes like aerobic glycolysis, fatty acid metabolism, and the utilization of tryptophan and glutamine (8). Since Warburg’s discovery of this phenomenon, the abnormal metabolism of cancer cells has been progressively studied (9). In addition, in ccRCC, this effect is shown to be more pronounced than in normal tissues, further highlighting its central role in tumour metabolism (10).

Glycometabolism in tumours is gradually becoming a hot topic of research, and higher levels of glycometabolism are one of the characteristics of tumour cells. A number of oncogenic factors may cause an increase in cellular glucose metabolism levels, leading to an increase in overall cellular metabolism levels (11). However, studies analysing the effects of glycometabolic genes on ccRCC based on the REACTOME database are still lacking, indicating that the roles and rationale of a large number of glycometabolic genes in the pathogenic mechanism of ccRCC are still not fully understood. Thus, we purpose to search the key sites of action of glucose metabolism and supply an important theoretical basis for further research. Through searching, we found as many as 89 genes associated with glucose metabolism in renal cancer, and we plan to identify the key genes associated with the prognosis of ccRCC through bioinformatics analyses and basic experimental validation, and to establish a prognostic prediction model.

**Figure 1:**
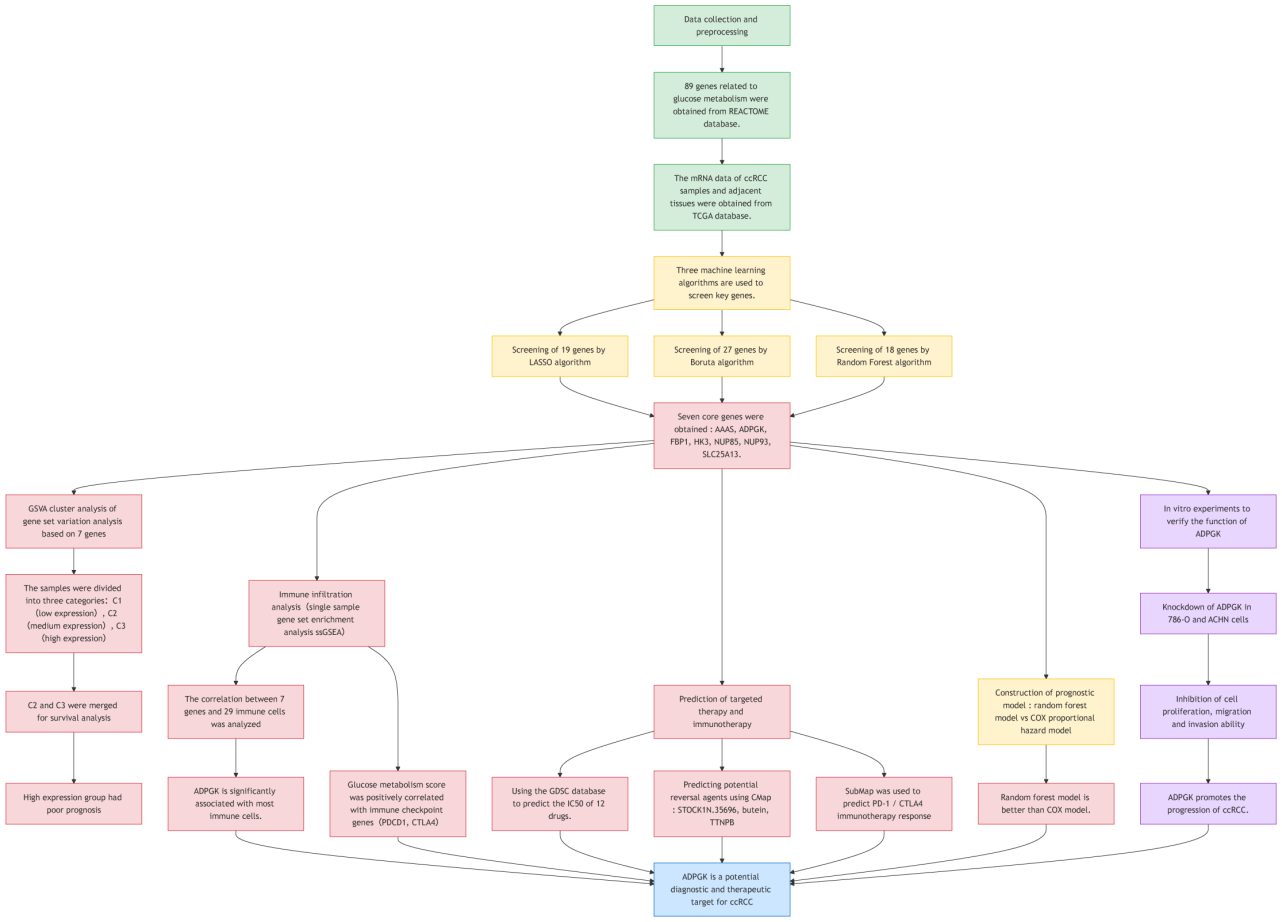
Flowchart of the entire article.

In this study, we used ccRCC samples (n=539) and corresponding paracancerous tissue samples (n=72) from the TCGA database to explore the role of glucose metabolism in ccRCC. To this end, we employed three machine learning algorithms: least absolute shrinkage and selection operator (LASSO), Boruta, and random forest (RF) to screen out the most robust targets, and ultimately discovered *ADPGK*, a glycometabolic target that has never been mentioned before in ccRCC. We further validated the effect of *ADPGK* on invasion and metastasis of ccRCC by cellular experiments, thus further revealing the relationship between glucose metabolism and ccRCC. A flowchart has been created to more clearly illustrate the experimental approach (**Figure 1**).

## 2 Materials and methods

### 2.1 Gather and analysis of sugar metabolism gene sets

A dataset related to glucose metabolism was obtained from the REACTOME database (12) (https://reactome.org/) according to the GSEA website and 89 genes closely related to glucose metabolism routes were chosen for deeply analyse. We retrieved the study data from the TCGA database (https://tcga-data.nci.nih.gov/tcga/) and screened the mRNA information of genes related to glucose metabolism in more than 30 cancers (13). Correlations between gene expression data were analysed using Perl.

### 2.2 Recognition of illness characterisation genes

For the characterization of illness diagnostic elements, we combined multiple machine learning methods, namely LASSO, Boruta and RF, to recognize key genes of the glucose metabolic pathway in ccRCC. LASSO and RF were used to regress survival time and survival state and thus screen categorical variables, LASSO regression model was built using “glmnet” package (14); RF model was constructed using “ randomForestSRC ” package (15). Risk score = ∑ Ni =1 (Expi∗ Coei); N, Coei and Expi denote the quantity of genes, regression correlation coefficients and gene expression levels achieved from LASSO regression analyse, separately. Screening of categorical variables based on best-supervised classification was performed using the Boruta algorithm, which was used to accurately screen all the genes that were most relevant for model prediction. Finally, the screened genes that performed well in all three machine learning algorithms simultaneously were selected (16).

### 2.3 Glucose metabolism score constructed by GSVA algorithm

GSVA is an analytical technique that determines the number and membership of possible clusters (microarray gene expression) between samples. Based on differences in characteristic gene expression (17). We employed the GSVA algorithm to estimate the glycometabolic pathway score in each TCGA sample, which reflects the level of enrichment of glycometabolic pathways in each sample and was used to determine whether they were highly, non-differently, or lowly expressed (halfwidth = 0.025), and which was used for subsequent immunoassays and drug sensitivity analyses of the targeted drugs. These samples were clustered using the Ward.D algorithm, with 1, 0, and -1 corresponding to C1 (low expression), C2 (non-differentiated), and C3 (high expression), respectively. Then, we created violin plots of characteristic gene accumulation scores in C1, C2, and C3 as well as survival curves after combining C2 and C3 to confirm the precision of the three clusters.

### 2.4 Estimation of immune cell infiltration

We employed ssGSEA analysis in conjunction with the TCGA database to measure the level of immune cell infiltration; their correlation outcomes are visualized as heatmaps. According to the ssGSEA outcomes, we show the interrelationships between 7 key genes for glucose metabolism and 29 immune cells, where the size of the sphere indicates the level of association. The R packages “ggStatsplot”, “data.table”, “ Tidyr”, “GGplot2” and “dplyr” were employed for statistical analyse of the data as well as for plotting heatmaps (18, 19). We then used the “ggdisterstats” package to generate scatter plots of the three most highly correlated immune factors: T cell co-inhibition, MHC class I and Type II IFN Reponse to show their correlation with glucose metabolism scores.

### 2.5 Prediction of targeted therapy and immunotherapy

We evaluated the efficacy of chemotherapy, including conventional ccRCC-targeted therapies and immunotherapies, in a patient population. To assess the response to chemotherapy in each individual, a ridge regression model was built based on TCGA gene expression profiles in the GDSC (https://www.cancerrxgene.org/) and using the R package “pRRohetic” to predict the IC50 of the drugs (20). The therapeutic correspondence of glycometabolically active subtypes with PD-1/CTLA-4 was revealed using “SubMap”, a dataset used to assess the effect of immunotherapy on different subtypes. The CMap algorithm provides us with several potential drugs that can reverse the molecular signature of glycometabolism, which are expected to be the reverse drugs for ccRCC (21).

### 2.6 Glucose metabolism mRNA screening reveals prognosis

Combining patients’ clinical information, glucose metabolism score subgroup information, and risk scores, we constructed both a random forest prognostic model and a COX regression prognostic model. With the help of the SURVEX software package, we compared the advantages and disadvantages of the two prognostic models and assessed the importance of each feature.

### 2.7 Statistical analyses

The statistical tasks of bioinformatics are performed by using R software (https://www.r-project.org/, version 4.4.1). For experiments, ImageJ and Graphpad Prism were chosen as the analysis and statistical software, which play a vital role in data processing and analysis.

### 2.8 Cell culture

The human renal cancer cell lines 786-O and ACHN and the normal renal proximal tubular epithelial cell line HK-2 were purchased from the Wuhan Pricella Biotechnology Co. ACHN cells were cultured in Dulbecco’s Modified Eagle’s/Eagle Medium (DMEM; Wuhan Pricella Biotechnology Co., Ltd.). 786-O cells were cultured in Roswell Park Memorial Institute medium (RPMI-1640; Wuhan Pricella Biotechnology Co., Ltd.).HK-2 cells were cultured in Minimum Essential Medium (MEM; Wuhan Pricella Biotechnology Co., Ltd.). All these cell lines were cultured in a medium containing 10% foetal bovine serum (FBS; Wuhan Pricella Biotechnology Co., Ltd.), 1% streptomycin-penicillin (Wuhan Pricella Biotechnology Co., Ltd.), and at 37 °C and 5% CO_2_.

### 2.9 siRNA transfection

Specifically targeting *ADPGK* (si-*ADPGK*) and negative control siRNA (si-NC) were obtained from Huzhou Hippo Biotechnology Co. In this research, the arrangement of *ADPGK* siRNA is presented below: 5’-GCUGAAUGAACAGGAGCUGUUTT-3’. For transfection, 786-O and ACHN cells were inoculated in 6-well plates at 50-60% confluency; 150 pmol of siRNA was confluent in 6ul in 6-well plates using GP-transfect-Mate transfection reagent (GenePharma, Inc.). 48 h after transfection, subsequent assays were performed.

### 2.10 RT-qPCR assay

cDNA was synthesised using the TRIGene Plus Total RNA Extraction Reagent and Auxiliary Kit (GenStar, Inc.) and StarScript ProAll-in-one RT Mix with gDNA Remover (GenStar, Inc.). Gene mRNA levels were analysed using the 2× RealStar Universal SYBR qPCR Mix Kit (GenStar, Inc.) following the manufacturer’s indications. The primer sequences are presented below: *ADPGK*, forward 5’-CCTAGAGCTGGGCCAGTATGACTA-3’, reverse 5’-GACTGGGGTGAGAAATAACAGCTC-3’. Calculated using the 2^ -ΔΔCt method.

### 2.11 Cell proliferation determination

Cell Counting Kit-8 (CCK-8; APExBIO Technology LLC) was used. Cells were inoculated into 96-well plates at a cell density of 2 x 10^3^ cells/well and cultured in 100 μL of cell culture medium containing 10% foetal bovine serum. Before measuring absorbance, 10 μL of CCK-8 Reagent and 100 μL of serum-free medium were added to each well to remove the effect of serum on the CCK-8 Reagent. After incubating the 96-well plate with CCK-8 Reagent for 2 hours at 37 °C and 5% CO2, the number of viable cells was evaluated by surveying the absorbance at 450 nm using a microplate reader. This was done every 24 hours for a total of 72 hours. Finally, cell numbers were plotted over a 3-day period using GraphPad Prism 9.1 (GraphPad Software, Inc.) to reflect the rate of cell proliferation.

### 2.12 Wound healing tests

For wound healing assays, cells were cultured in 6-well plates until they grow to complete confluence. Cell monolayers were scraped with a 200 μL pipette tip to form wounds. A light microscope equipment was used to acquire typical images of cell migration.

### 2.13 Cell migration and invasion assays

We used the Transwell assay to detect cell migration and invasion as described previously (22). A Transwell chamber containing an 8 μm membrane filter was used (Labselect, Inc.). Serum-free medium with 2 × 10^4^ cells/well was inoculated into the upper chamber, while the lower chamber was filled with medium containing 10% foetal bovine serum. After 48 hours of incubation at 37 °C, cells in the lower chamber were fixed with 4% paraformaldehyde fixative for 20 minutes at room temperature and then placed in crystal violet stain (Beyotime; C0121) for 30 minutes at room temperature. Finally, 3 random fields of view were counted under a light microscope at 100× magnification. For the invasion assay, Matrigel (Abwbio, Inc.) was pre-coated into the upper chamber for 3 hours. Cells (5×10^4^) were then inoculated into the upper chamber in serum-free medium. The remaining experimental steps were the same as for the migration assay.

### 2.14 Western blot assay

Western blot analysis was performed to evaluate the differential expression of ADPGK proteins in tumour and paracancerous tissues. Protein samples are separated using sodium dodecyl sulfate-polyacrylamide gel electrophoresis and transferred to a polyvinylidene difluoride (PVDF) membrane. PVDF membranes were closed with 5% skimmed milk in a shaker for 2 hours at 37 °C and incubated with ADPGK antibody, (#15639-1-AP, working dilution 1:1000, proteintech) incubated overnight at 4 °C. After 3 washes in TBST buffer solution for 30 minutes, the membrane was incubated with HRP-coupled goat anti-rabbit IgG H&L secondary antibody (#AS014, working dilution 1:10000, ABclonal) for 1.5 hours. Then wash with TBST buffer solution 3 times for 30 minutes each time. Results were analysed using the Ultra Sensitive ECL Chemiluminescence Kit (SW134-01; Sevenbio). Immunoblotting was quantified using Image J software.

## 3 Results

### 3.1 Machine learning algorithm to identify target gene ADPGK

**Figure 2:**
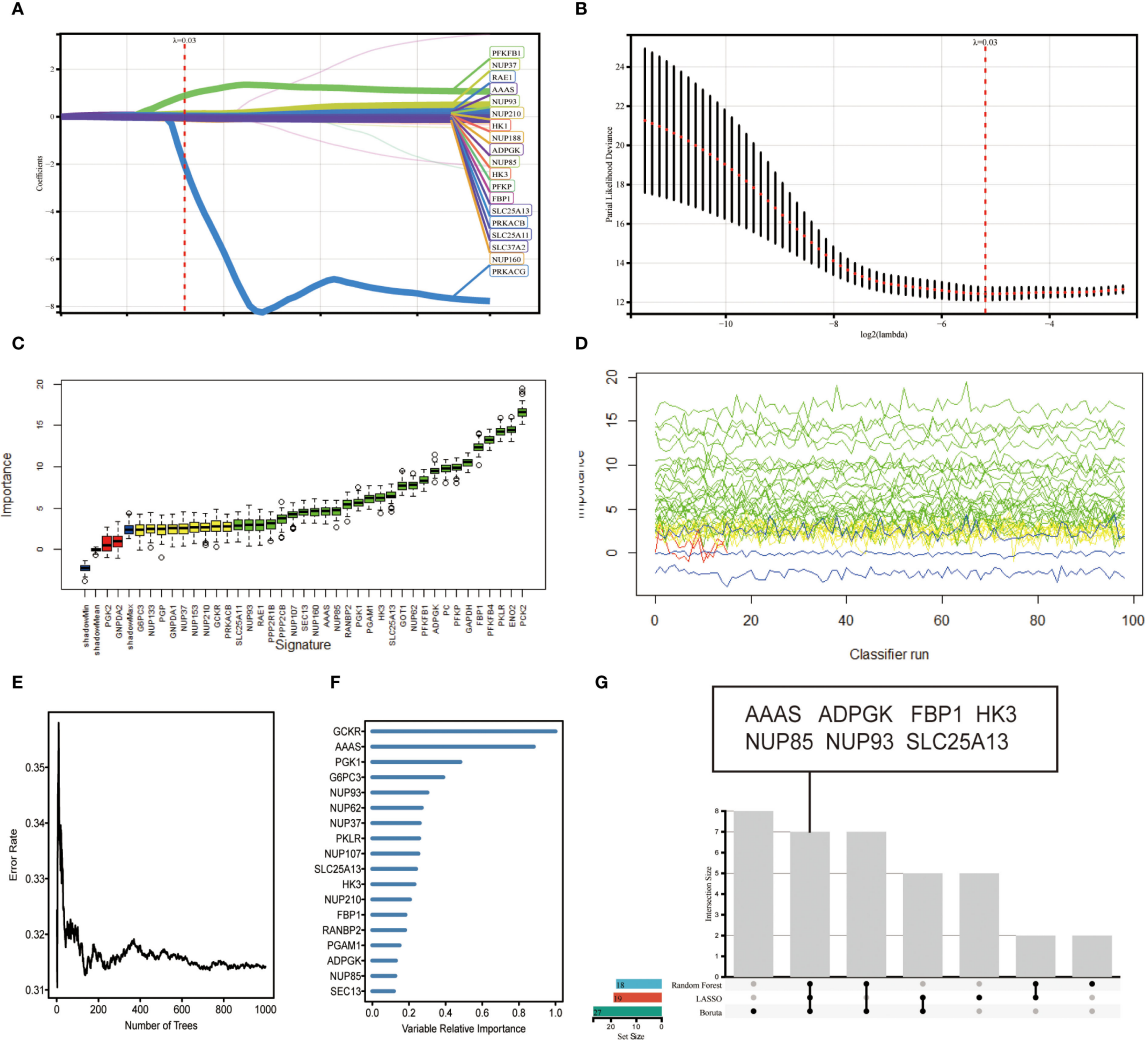
Variable screening based on LASSO algorithm, Boruta algorithm, RF algorithm. **(A)** Characteristics of variation of variable coefficients. **(B)** Selection process of optimal value of parameter λ in LASSO regression model by cross-validation method. **(C)** Boruta selected 27 typical genes with importance ranking. **(D)** Plot of the variation of Z-scores. **(E)** Error rate of data as a function of classification tree. **(F)** Random survival forest analysis identified 18 core genes. **(G)** upset plot showing that 7 candidate feature genes *AAAS*, *ADPGK*, *FBP1*, *HK3*, *NUP85*, *NUP93*, *SLC25A13* were identified by the above 3 machine learning algorithms.

The 89 glucose metabolism-related genes contained in REACTOME of the GSEA database were used for gene screening. A combination of three machine learning methods, LASSO algorithm, Boruta algorithm, and RF algorithm, was used to locate the genes at the kernel of the research. By using LASSO algorithm to screen for the correlation between glucose metabolism bases and CCRCC, 19 genes that could serve as potential markers were identified (**Figure 2A**). A 10-fold cross-validation method was applied to iterative analysis, and when λ was 0.03 (Log2 λ = -5.058), 19 genes were screened to obtain a model with excellent performance but the lowest number of variables (**Figure 2B**). The Boruta algorithm analysis screened 27 significant genes, which showed significantly elevated AUCs in the model, suggesting that they had a strong influence on the outcome variable (**Figure 2C**). The line graph demonstrates the screening process based on parameter variations (**Figure 2D**). To further identify the core genes in glucose metabolism genes that affect the prognosis of CCRCC, we performed RF analysis on these 89 genes, and we ranked the genes according to their importance in the RF model, identified the genes with relative importance as the final markers, and finally screened out 18 key genes (**Figure 2F**). The intersection of the results of the three machine learning methods was taken, and 7 key genes achalasia (*AAAS*), *ADPGK*, fructose-1,6-bisphosphatase 1(*FBP1*), hexokinase 3(*HK3*), nucleoporin 85kDa (*NUP85*), nucleoporin 93kDa (*NUP93*), and solute carrier family 25, member 13 (*SLC25A13*) were finally screened (**Figure 2G**). Among them *AAAS*, *HK3*, and *ADPGK* the expression of three genes was obviously raised in cancer samples and was significantly correlated with prognosis. Our research is also the first to study the characterization of glucose metabolism-related genes in REACTOME in ccRCC by machine learning.

### 3.2 These three clusters correspond to the expression of genes related to sugar metabolism

**Figure 3:**
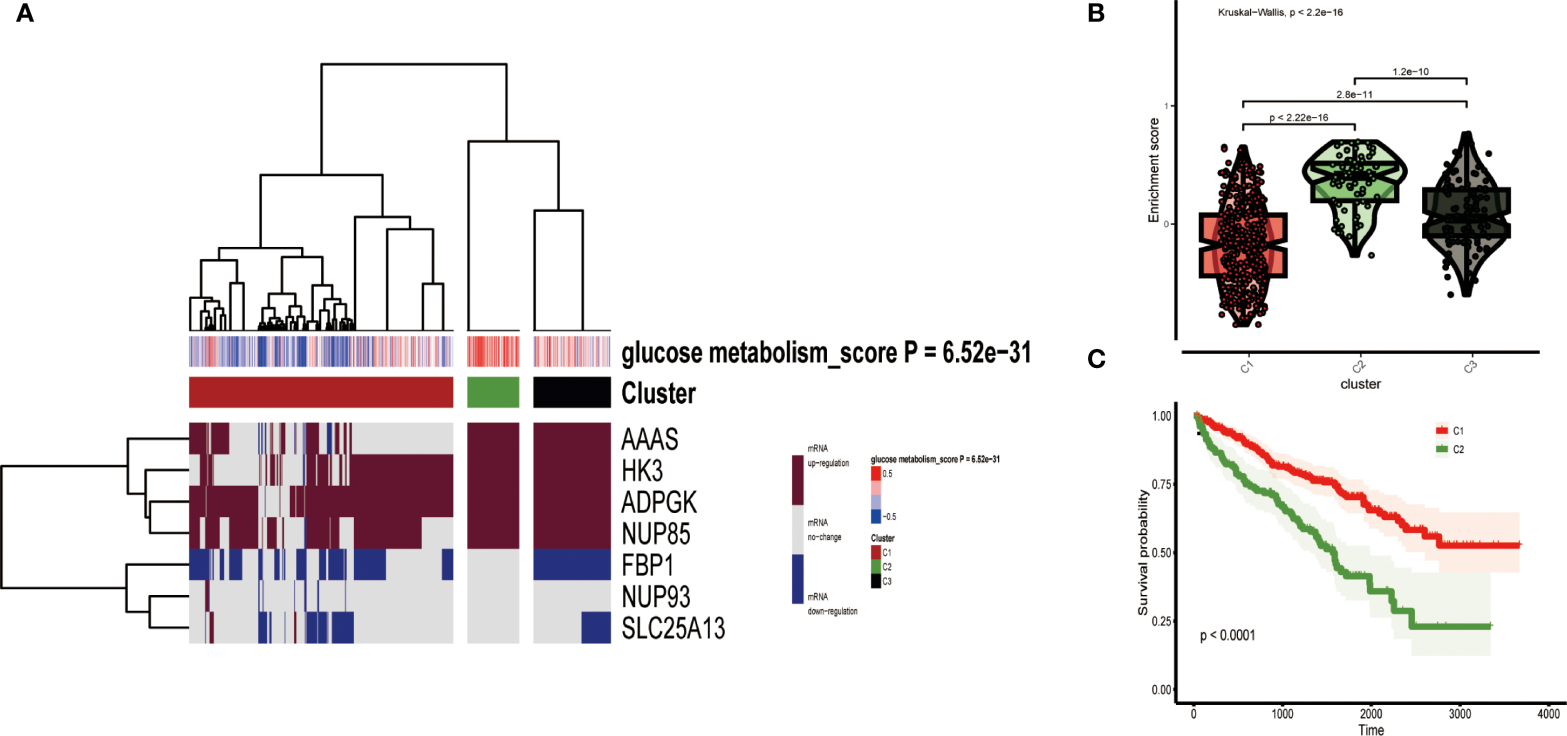
**(A)** All ccRCC samples were divided into three groups according to different levels of glucose metabolism scores: low expression group (C1), high expression group (C2) and medium expression group (C3). Colour changes in the right colour bar indicate different values: dark red indicates up-regulation of mRNA expression and dark blue indicates down-regulation of mRNA expression. The closer the glucose metabolism score is to 0.5 the redder the colour is, the closer it is to -0.5 the bluer the colour is. The three clusters formed by cluster analysis are represented by different colours: red for C1, olive green for C2, and black for C3. **(B)** Violin plots showing the accumulation scores of the three clusters. **(C)** Survival curves based on the merged C2 versus the two clusters of the original C1 group.

Based on the mRNA expression of 7 key genes for glucose metabolism combined with the GSVA algorithm, unsupervised cluster analyses was applied to acquire three clusters of TCGA data from ccRCC: low expression cluster (C1), high expression cluster (C2), and medium expression cluster (C3) (**Figure 3A**). The heatmap showed that the mRNA expression level of C1, symbolizing low expression of gluconeogenesis, was universally downregulated, and the mRNA expression of C2, symbolizing high expression of gluconeogenesis, was significantly upregulated. The accumulation scores of glucose metabolism-related genes in the three clusters confirmed that the levels of glucose metabolism-related genes were low in C1 and obviously upper in C2 than in C1 (**Figure 3B**). Afterwards, we merged C2 and C3 and performed survival prognosis analysis and plotted survival curves for the merged C2 versus the original C1. The survival curves showed that C2 with higher glucose metabolism expression levels had a significantly worse prognosis, while C1 with low glucose metabolism expression levels had a good prognosis, and there was also a large difference between the survival curves of the two clusters of merged C2 and the original C1, which proved the significance of our merger (**Figure 3C**).

### 3.3 Analysis of immune infiltration and immune checkpoint blockade

**Figure 4:**
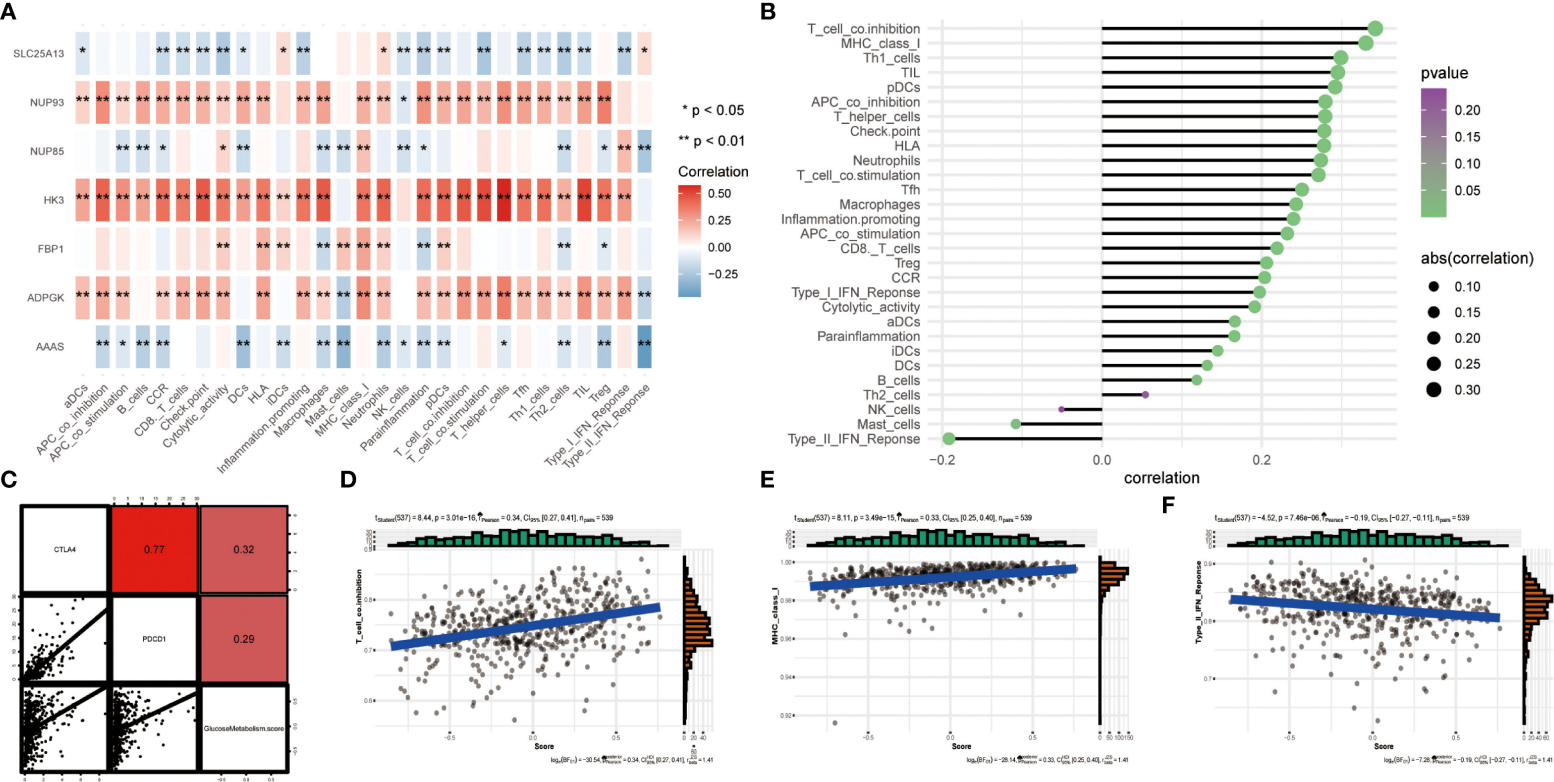
**(A)** Heatmap showing the correlation between the 7 glucose metabolism related genes and various immune infiltration related metrics. The colour bars on the right show that the closer to red, the greater the positive correlation, and the closer to blue, the greater the negative correlation. *represents *P* < 0.05 and ** represents *P* < 0.01. **(B)** Bubble plots show the degree of correlation. Bubble size indicates the magnitude of correlation from 0.1 to 0.3, and colour indicates the p-value from 0 to 0.25. **(C)** Scatter plot showing the correlation between glucose metabolism and PDCD1, CTLA4. **(D-F)** Three scatter plots showing the correlation between glucose metabolism scores and T cell co-inhibition, MHC class I and Type II IFN Reponse, respectively.

In recent years, apart from studying the role of targeted drugs in cancer treatment, there has been an increasing number of studies on immunotherapy (23). We investigated the quantitative correlation between immune cell infiltration and 7 key genes of glucose metabolism, which can reflect the regulatory role of glucose metabolism pathway in ccRCC immunotherapy. The heatmap showed the quantitative correlation between immune cell infiltration and the 7 key genes of glucose metabolism (**Figure 4A**), from which the results showed that majority of the immune cells were actively related with *ADPGK* with a statistically significant difference. Bubble plots indicated an association between immune infiltration-related cells or features and the 7 glucose metabolism key genes (**Figure 4B**). Subsequently, we verified the correlation between glucose metabolism scores and immune checkpoint-related genes, and the results showed that the glucose metabolism scores were positively correlated with the levels of PDCD1 and CTLA4, which indicated that immunotherapy was feasible on the glucose metabolism pathway (**Figure 4C**). In addition, we selected the first two immune factors with positive correlation, T cell co-inhibition, MHC class I, and one negative correlation, immune function Type II IFN Reponse, for association analyses, and the outcomes showed that their correlation with glucose metabolism and bubble plots showed the same trend (**Figures 4D-F**).

### 3.4 Predictive model analysis and validation

**Figure 5:**
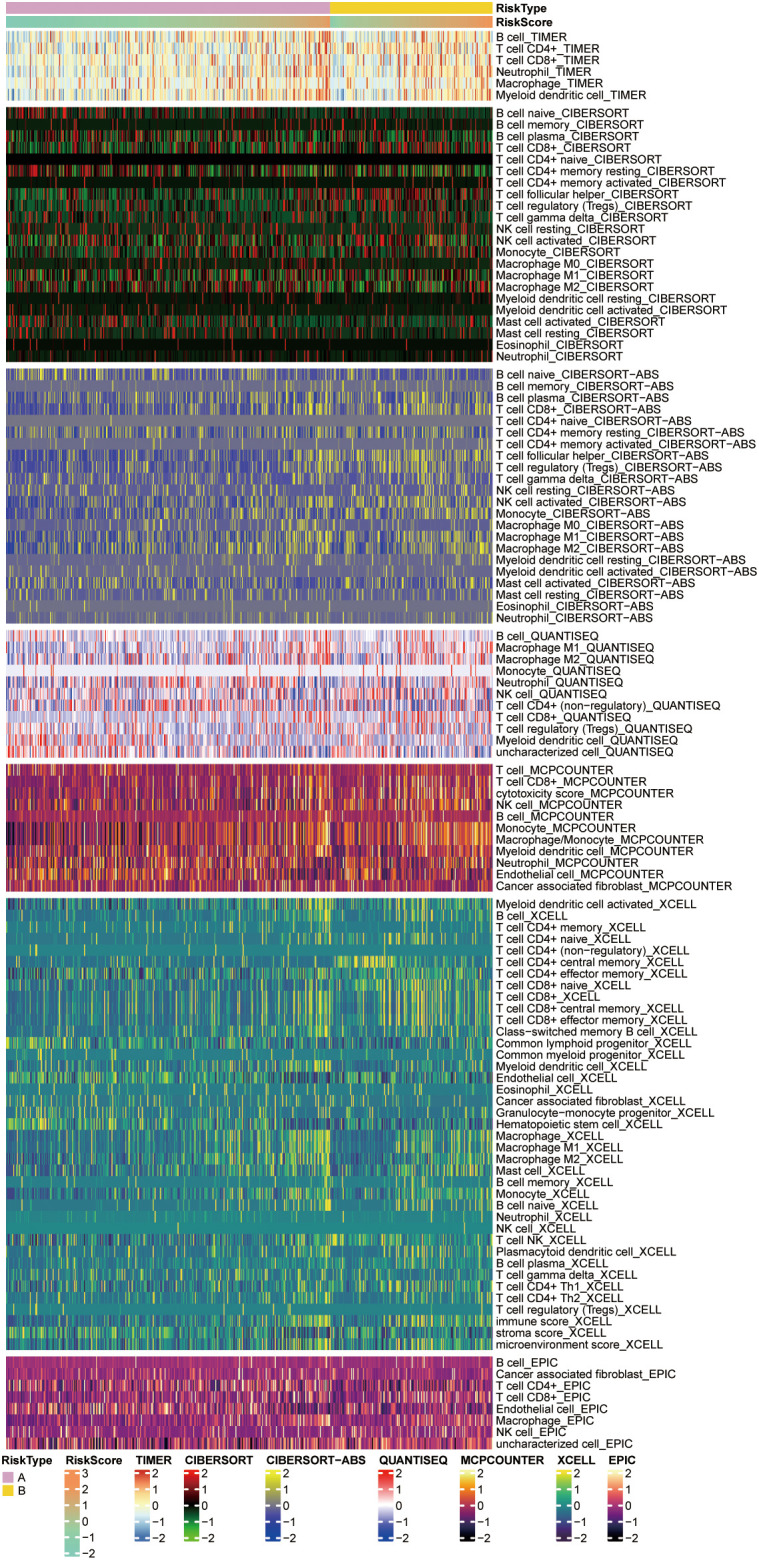
Heatmaps showing immune cell responses in the high and low risk groups based on different algorithms. Pink and yellow indicate high and low penetration levels, respectively, and different algorithms are indicated by different coloured area bars.

Based on the 7 previously selected key genes for glucose metabolism, *AAAS*, *ADPGK*, *FBP1*, *HK3*, *NUP85*, *NUP93*, and *SLC25A13*, we computed risk scores for the data. Based on the risk score values acquired, the data were separated into two groups, high-risk score and low-risk score, and their correlation with the extent of immune cell infiltration was compared employing diverse immune infiltration algorithms, like the XCELL algorithm and the TIMER algorithm for the low expression of CD8+ T-cells in the high-risk score (**Figure 5**). These outcomes can be employed to study the differences in the immune microenvironment between patients with different ccRCC and to explore the therapeutic effect of immunotherapy in practical applications.

### 3.5 Predictive analysis of immunotherapy

**Figure 6:**
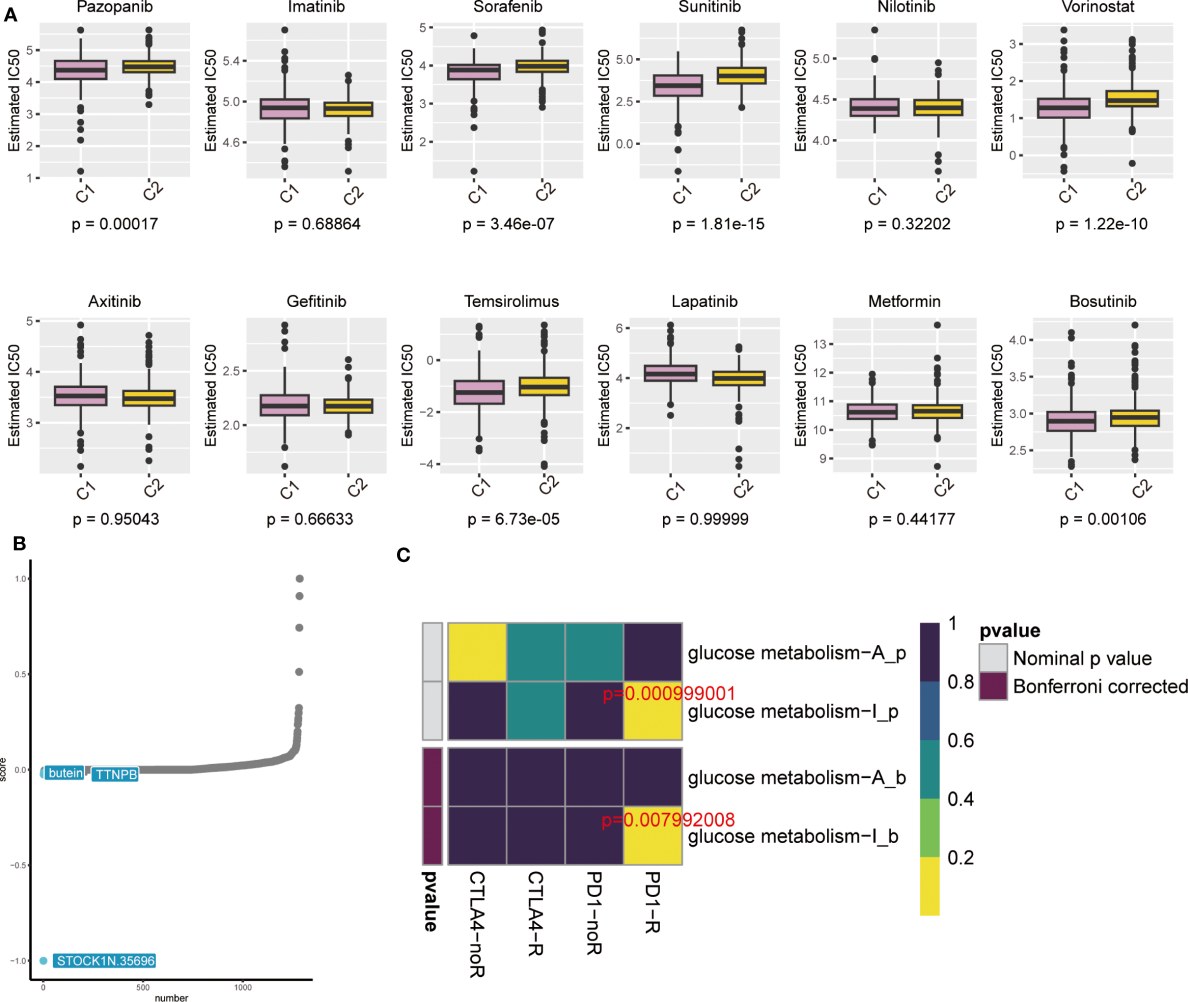
**(A)** Box line plot showing IC50 prediction of KIRC cells treated with common tumour-targeting drugs. **(B)** The cMAP algorithm predicts small molecule drugs that may have an effect on the gluconeogenic pathway. **(C)** Heatmap showing predictions of p-values obtained by comparing glycometabolically active and non-glycometabolically active samples using PD-1 and CTLA4 treatments, and comparative results of p-values after Bonferroni correction.

For the anticipation of pharmacological experimental outcomes in patients with ccRCC, the Genomics of Drug Sensitivity in Cancer (GDSC) database is critical. Depending on the expression of genes in different cell lines in the GDSC database and supported by the pRRophetic algorithm, we anticipated the pharmacological influences of ccRCC cells on 12 common first- or second-line oncological chemotherapeutic and targeted agents used in the clinic: pazopanib, sorafenib, sunitinib, nilotinib, vorinostat, acitretinib, gefitinib, ticlosimabe, lapatinib, metformin, bosutinib and tipifarnib. The outcomes of the drug IC50 anticipation analyses indicated that the anticipated IC50 values of most of the targeted drugs for combined C2 were obviously less than that of C1, suggesting that patients with ccRCC who have high expression of glucose metabolism-related genes would be more susceptible to these conventionally targeted drugs (**Figure 6A**). Therefore, these targeted drugs are of particular importance for the therapy of patients with high expression of glucose metabolism-related genes in ccRCC. From the outcomes, we noticed that currently common cancer-targeted drugs in the clinic: axitinib (24), gefitinib (25)and lapatinib (26)are highly sensitive to ccRCC patients with high expression of glucose metabolism-related genes, which also suggests that the glucose metabolism pathway may be instructive for the development of ccRCC-targeted drugs. In addition, we used the CMap algorithm to predict small-molecule drugs that may have an effect on the glucose metabolism pathway. The results showed that STOCK1N.35696, butein, and TTNPB were the top three potential drugs for treating ccRCC patients with high expression of glucose metabolism genes (**Figure 6B**). The heatmap indicated that the reaction to immune checkpoint Programmed cell death protein 1(PD-1) treatment was statistically significant in both the pre-calibration (*p* = 0.000999001) as well as the post-calibration (*p* = 0.007992008) glucose metabolism activity groups (**Figure 6C**). This suggests that patients with high expression of glucose metabolism-related genes in ccRCC may be responsive to immune checkpoint inhibitors and that PD-1 therapy holds promise for development in these patients.

### 3.6 Silencing of the ADPGK gene attenuates glycolysis and promotes cell death in 786-O and ACHN cells

**Figure 7:**
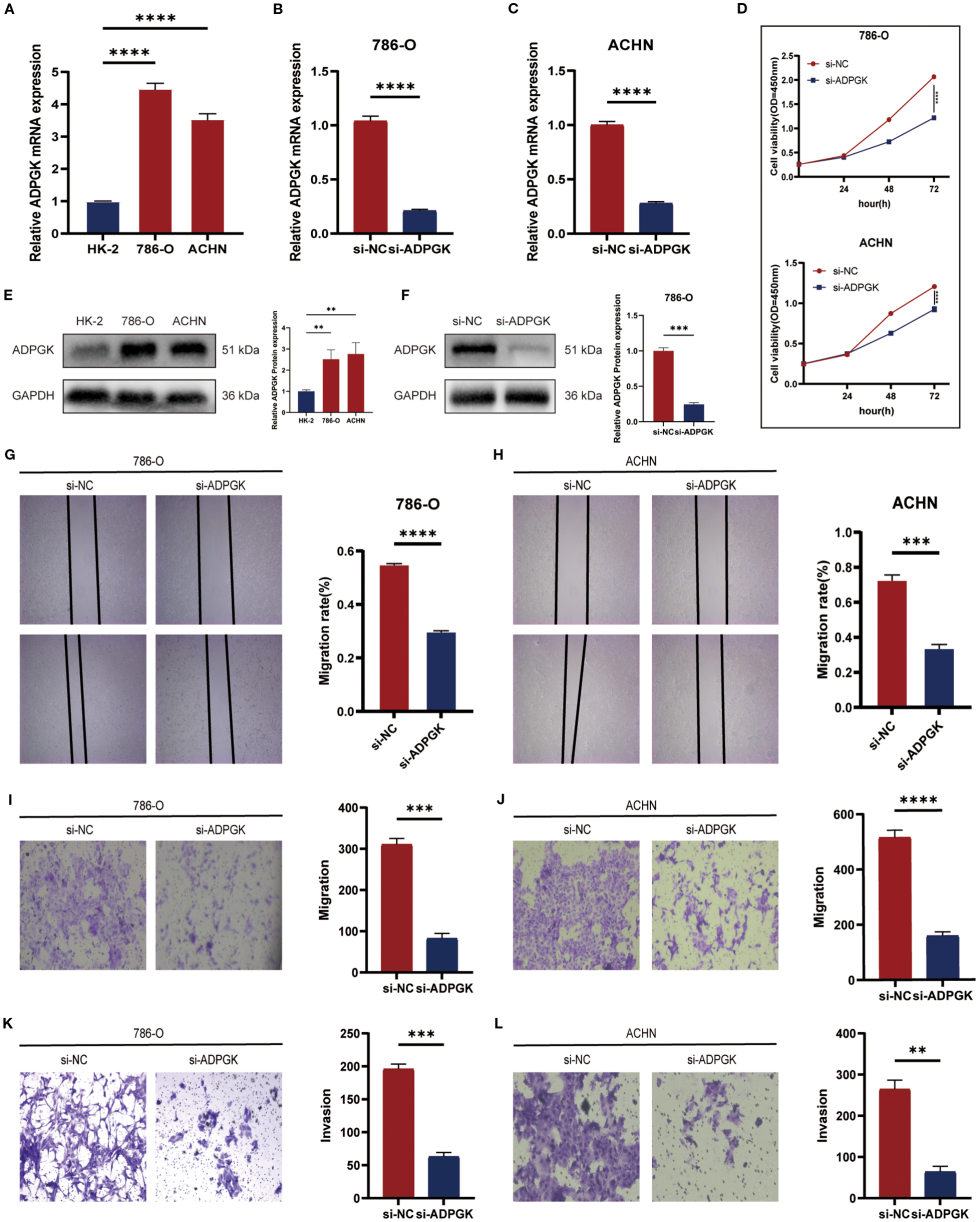
**(A-C)** qPCR showing the comparison of mRNA expression of *ADPGK* between 786-O and ACHN cells and HK-2 cells and the efficiency of knocking down *ADPGK* on 786-O and ACHN cell lines. **(D)** CCK-8 assay showing the viability of cell proliferation after knocking down *ADPGK* compared to the non-knockdown group. **(E)** WB displays a comparison of ADPGK protein expression between 786-O and ACHN cells and HK-2 cells. **(F)** WB showed the protein expression changes after knocking down ADPGK in the 786-O cell line. **(G-J)** Wound healing assay and Transwell migration assay showed the migration ability of 786-O and ACHN cells treated with si-NC or si-*ADPGK*. **(K, L)** Transwell invasion assays showing the invasion ability of 786-O and ACHN cells treated with si-NC or si-*ADPGK*. Data are expressed as SD ± mean. *P < 0.05, **P < 0.01, ***P < 0.001, ****P < 0.0001.

To assess the biological role of *ADPGK* in ccRCC, small interfering RNAs (siRNAs) specifically targeting *ADPGK* binding were designed. *ADPGK* RNA expression was validated in several ccRCC cell lines. qPCR results showed that *ADPGK* expression was relatively high in 786-O and ACHN cells compared to the normal renal proximal tubular epithelial cell line HK-2, consistent with the above (**Figure 7A**). Transfection of 786-O and ACHN cells with *ADPGK* siRNA for 48 h showed that *ADPGK* was successfully knocked down (**Figures 7B, C**). CCK-8, colony formation (**Supplementary Figure S1**) assay showed that down-regulation of *ADPGK* inhibited the proliferative activity of 786-O and ACHN cells as compared to the si-NC group (**Figure 7D**). In addition, we verified the protein expression and knockdown efficiency of ADPGK through WB experiments. The results showed that the expression of ADPGK in 786-O and ACHN cells was relatively higher compared with that in normal human proximal tubular epithelial cell line HK-2, which was consistent with the above (**Figure 7E**). The WB results indicated that ADPGK was successfully knocked down (**Figure 7F**). Transwell migration assay and wound healing assay showed that silencing of *ADPGK* significantly inhibited the migration of 786-O and ACHN cells (**Figures 7G-J**). Transwell invasion assay showed that the invasive ability of 786-O and ACHN cells was significantly reduced after knockdown of *ADPGK* (**Figures 7K, L**). These results suggest that the *ADPGK* gene is a key therapeutic target located on the ccRCC glucose metabolism pathway.

### 3.7 Machine learning-based prognostic judgement for ccRCC patients

**Figure 8:**
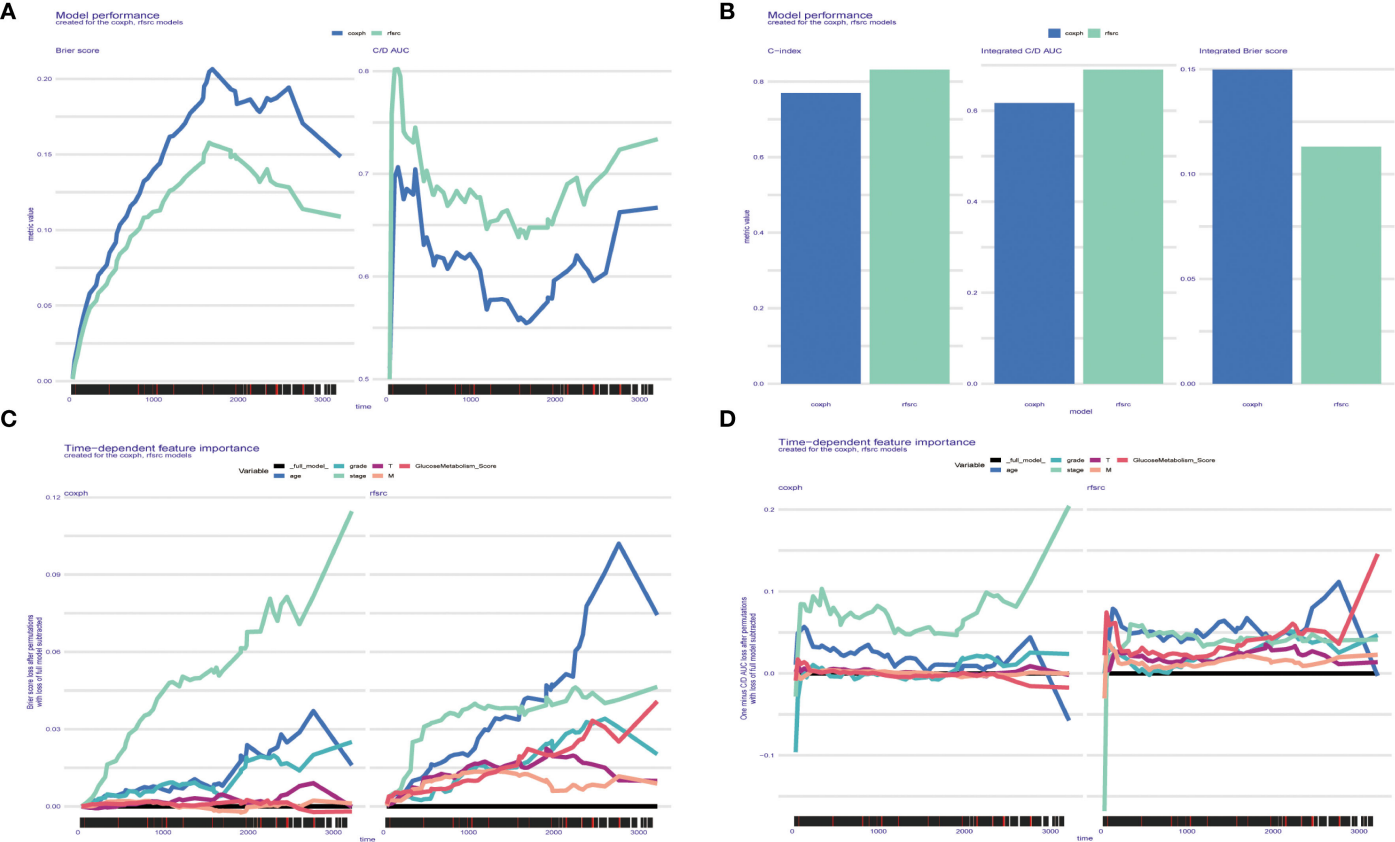
**(A-D)** Glucose metabolism scores obtained based on the SURVEX package, constructing both the random forest model and the COX regression model of the MOMC-VM and combining it with machine learning.

According to the Brier score and the C/D AUC index based on survival time, it can be found that the Brier score value of random survival forest (rfsrc) is always lower than that of Cox proportional hazards (coxph), while the C/D AUC value is always higher than that of coxph, which indicates that rfsrc’s predictive power is better than coxph, and the C-index results are consistent with the previous two results (**Figures 8A, B**). The line graph of time-dependent feature importance shows that in coxph, the importance of Stage increases with time, while in rfsrc, GlucoseMetabolism Score shows an increase in importance with time, which is consistent with our previous findings (**Figures 8C, D**).

## 4 Discussion

Glucose metabolism, including glycolysis, glycogen synthesis, glycogenolysis and gluconeogenesis, is an important physiological process in the maintenance of metabolic homeostasis in organisms, and plays a key role in the growth of tumour cells (4). Among them, glycolysis is the main way to break down glucose into pyruvate and generate ATP. In tumour cells, this phenomenon is known as the “Warburg effect^”^ (27). However, even under well-oxygenated conditions, the source of ATP production in tumour cells is not aerobic oxidation, but rather glycolysis (28). Lactate, a metabolite of aerobic glycolysis, promotes tumour cell growth and metastasis through several pathways (29). Glycogen synthesis is the process by which excess carbohydrates are converted to glycogen or fatty acids under high-sugar dietary conditions and stored in the liver (30). In contrast, glycogenolysis is activated under conditions of energy deficiency to release glucose to meet the energy needs of tissues such as the brain (31). However, when hepatic glycogen reserves are depleted during prolonged energy deprivation, glucose is rapidly synthesised via gluconeogenesis, a process that typically utilises substances such as lactate, glycerol and amino acids as substrates (32). Glucose metabolism not only plays a key role in maintaining normal renal cell survival, but is also critical for ccRCC survival (33). Therefore, targeting glucose metabolism to kill ccRCC cells may be a potential therapeutic option.

In our study, we first used a combination of three machine learning algorithms, LASSO algorithm, Boruta algorithm, and RF algorithm, to screen the 7 core glucose metabolism genes of the study, *AAAS*, *ADPGK*, *FBP1*, *HK3*, *NUP85*, *NUP93*, and *SLC25A13*. Among them, LASSO is one of the first machine learning algorithms used and is suitable for handling data with multiple covariates, but may not handle some nonlinear features completely (34). The RF model allows random sampling of the original features thus generating random features and calculating feature importance (35). The Boruta algorithm is based on the RF model and filters out more significant nonlinear features by comparing the importance of raw and random features (16). The combination of these three algorithms helps to improve the accuracy of feature screening, more comprehensively identifies key genes for glucose metabolism associated with ccRCC, and reduces the bias that may result from a single approach. Then, based on the mRNA expression of the 7 glucose metabolism-related genes in combination with the GSVA algorithm to obtain three clusters of ccRCC samples. Based on the two clusters, C1, which is the low expression of glycometabolic genes, and C2, which is the combination of medium and high expression of glycometabolic genes, we constructed the survival curves of glycometabolism-related genes. In the survival curves, we noted that upregulation of glycometabolic genes had the lowest survival values, suggesting that most glycometabolism-related genes are risk genes in ccRCC that promote tumour cell growth. To explore the role of targeted therapy as well as immunotherapy in ccRCC, we further analysed the targeted drug and immunotherapy predictions, and from the results, we observed that targeted drug therapy in ccRCC was closely associated with changes in gene expression levels in the glucose metabolism pathway. After clarifying the correlation between immune cells and glucose metabolism genes, we verified the correlation of glucose metabolism scores with the immune checkpoint genes PDCD1 and CTLA4 and compared the p-value predictions of two clusters of glucose metabolism scores after treatment with PD-1 and Cytotoxic T lymphocyte-associated antigen-4 (CTLA-4). The results suggest that glucose metabolism is important in tumour immunotherapy, which is uniform with former findings (36). These findings provide new ideas for the development of immunotherapies as well as drugs targeted for the treatment of ccRCC.

Subsequently, we chose *ADPGK* as a follow-up study gene. The results of *in vitro* experiments showed that *ADPGK* is significantly important for ccRCC cell proliferation and migration, and it would promote the proliferation and migration of ccRCC cells. ccRCC CPTAC database samples results further demonstrated that *ADPGK* was obviously extremely expressed and the upper the expression of *ADPGK*, the inferior the prognosis of ccRCC. *ADPGK*, also known as ADP-dependent glucose kinase, is an enzyme involved in the glycolytic pathway. Unlike typical ATP-dependent hexokinases (HKs), it catalyses the phosphorylation of glucose to glucose 6-phosphate using ADP rather than ATP as the phosphate donor (37). The role of *ADPGK* on the growth of ccRCC is unclear and few studies have been conducted. 2012, Richter et al. showed that silencing or overexpression of *ADPGK* in lung cancer cells did not affect anaerobic glycolysis but reduced clone formation in lung cancer cells (38). 2023, Xu et al. studied the expression and role of *ADPGK* in prostate cancer cells in *in vitro* and *in vivo* experiments, and their results proved that overexpression of *ADPGK* promotes a malignant phenotype, and inhibition of *ADPGK* suppresses the proliferation and migration of prostate cancer cells. In addition, their experiments demonstrated that *ADPGK* could promote glycolysis in prostate cancer cells through activation of the ALDOC-AMPK pathway (39). Due to the high degree of heterogeneity between different tumours, it is uncertain whether these two contradictory conclusions apply to ccRCC and whether *ADPGK* can adjust glycolysis in ccRCC cells. Our research affords preliminary proof that *ADPGK* is a driver of ccRCC progression and that its high expression leads to a poor prognosis in ccRCC patients.

In order to explore better prognostic models, this study constructed the rfsrc model, which outperformed the previously routinely used coxph in terms of predictive power and had more favourable predictive power for external data. In addition, the rfsrc predictions showed that the importance of glucose metabolism scores increased with time, which is consistent with our findings.

## Data Availability

The datasets presented in this study can be found in online repositories. The names of the repository/repositories and accession number(s) can be found in the article/**Supplementary Material**.

## Supplementary material

Supplementary Figure S1 Colony formation assay.

## References

[1] Siegel RL Miller KD Jemal A. Cancer statistics, 2017. CA: Cancer J Clin. (2017) 67:7–30. doi: 10.3322/caac.21387, PMID: 28055103

[2] Gupta K Miller JD Li JZ Russell MW Charbonneau C. Epidemiologic and socioeconomic burden of metastatic renal cell carcinoma (mRCC): a literature review. Cancer Treat Rev. (2008) 34:193–205. doi: 10.1016/j.ctrv.2007.12.001, PMID: 18313224

[3] Makhov P Joshi S Ghatalia P Kutikov A Uzzo RG Kolenko VM. Resistance to systemic therapies in clear cell renal cell carcinoma: mechanisms and management strategies. Mol Cancer Ther. (2018) 17:1355–64. doi: 10.1158/1535-7163.MCT-17-1299, PMID: 29967214 PMC6034114

[4] Zhu J Thompson CB. Metabolic regulation of cell growth and proliferation. Nat Rev Mol Cell Biol. (2019) 20:436–50. doi: 10.1038/s41580-019-0123-5, PMID: 30976106 PMC6592760

[5] Koppenol WH Bounds PL Dang CV. Otto Warburg’s contributions to current concepts of cancer metabolism. Nat Rev Cancer. (2011) 11:325–37. doi: 10.1038/nrc3038, PMID: 21508971

[6] Liberti MV Locasale JW. The warburg effect: how does it benefit cancer cells? Trends Biochem Sci. (2016) 41:211–8. doi: 10.1016/j.tibs.2016.01.004, PMID: 26778478 PMC4783224

[7] Wettersten HI Aboud OA Lara PN Jr. Weiss RH. Metabolic reprogramming in clear cell renal cell carcinoma. Nat Rev Nephrol. (2017) 13:410–9. doi: 10.1038/nrneph.2017.59, PMID: 28480903

[8] Lucarelli G Loizzo D Franzin R Battaglia S Ferro M Cantiello F. Metabolomic insights into pathophysiological mechanisms and biomarker discovery in clear cell renal cell carcinoma. Expert Rev Mol Diagn. (2019) 19:397–407. doi: 10.1080/14737159.2019.1607729, PMID: 30983433

[9] Wang G Wang JJ Guan R Du L Gao J Fu XL. Strategies to target glucose metabolism in tumor microenvironment on cancer by flavonoids. Nutr Cancer. (2017) 69:534–54. doi: 10.1080/01635581.2017.1295090, PMID: 28323500

[10] Courtney KD Bezwada D Mashimo T Pichumani K Vemireddy V Funk AM. Isotope tracing of human clear cell renal cell carcinomas demonstrates suppressed glucose oxidation *in vivo* . Cell Metab. (2018) 28:793–800.e2. doi: 10.1016/j.cmet.2018.07.020, PMID: 30146487 PMC6221993

[11] D’Souza LC Shekher A Challagundla KB Sharma A Gupta SC. Reprogramming of glycolysis by chemical carcinogens during tumor development. Semin Cancer Biol. (2022) 87:127–36. doi: 10.1016/j.semcancer.2022.10.004, PMID: 36265806

[12] Jassal B Matthews L Viteri G Gong C Lorente P Fabregat A. The reactome pathway knowledgebase. Nucleic Acids Res. (2020) 48:D498–d503. doi: 10.1093/nar/gkz1031, PMID: 31691815 PMC7145712

[13] Tomczak K Czerwińska P Wiznerowicz M. The Cancer Genome Atlas (TCGA): an immeasurable source of knowledge. Contemp Oncol (Poznan Poland). (2015) 19:A68–77. doi: 10.5114/wo.2014.47136, PMID: 25691825 PMC4322527

[14] Engebretsen S Bohlin J. Statistical predictions with glmnet. Clin Epigenet. (2019) 11:123. doi: 10.1186/s13148-019-0730-1, PMID: 31443682 PMC6708235

[15] Ishwaran H LM Kogalur UB. Randomforestsrc: variable importance (VIMP) with subsampling inference vignette. (2021).

[16] Kursa MB Rudnicki WR. Feature selection with the boruta package. J Stat Softw. (2010) 36(11):1–13. doi: 10.18637/jss.v036.i11

[17] Hänzelmann S Castelo R Guinney J. GSVA: gene set variation analysis for microarray and RNA-seq data. BMC Bioinf. (2013) 14:7. doi: 10.1186/1471-2105-14-7, PMID: 23323831 PMC3618321

[18] Román Palacios C Wright A Uyeda J. treedata.table: a wrapper for data.table that enables fast manipulation of large phylogenetic trees matched to data. PeerJ. (2021) 9:e12450. doi: 10.7717/peerj.12450, PMID: 34900417 PMC8628621

[19] Ito K Murphy D. Application of ggplot2 to pharmacometric graphics. CPT: Pharmacometrics Syst Pharmacol. (2013) 2:e79. doi: 10.1038/psp.2013.56, PMID: 24132163 PMC3817376

[20] Yang W Soares J Greninger P Edelman EJ Lightfoot H Forbes S. Genomics of Drug Sensitivity in Cancer (GDSC): a resource for therapeutic biomarker discovery in cancer cells. Nucleic Acids Res. (2013) 41:D955–61. doi: 10.1093/nar/gks1111, PMID: 23180760 PMC3531057

[21] Gao Y Kim S Lee YI Lee J. Cellular stress-modulating drugs can potentially be identified by in silico screening with connectivity map (CMap). Int J Mol Sci. (2019) 20:5601. doi: 10.3390/ijms20225601, PMID: 31717493 PMC6888006

[22] Yu-Ju Wu C Chen CH Lin CY Feng LY Lin YC Wei KC. CCL5 of glioma-associated microglia/macrophages regulates glioma migration and invasion via calcium-dependent matrix metalloproteinase 2. Neuro-oncology. (2020) 22:253–66. doi: 10.1093/neuonc/noz189, PMID: 31593589 PMC7032635

[23] Meng L Collier KA Wang P Li Z Monk P Mortazavi A. Emerging immunotherapy approaches for advanced clear cell renal cell carcinoma. Cells. (2023) 13:34. doi: 10.3390/cells13010034, PMID: 38201238 PMC10777977

[24] Karam JA Devine CE Urbauer DL Lozano M Maity T Ahrar K. Phase 2 trial of neoadjuvant axitinib in patients with locally advanced nonmetastatic clear cell renal cell carcinoma. Eur Urol. (2014) 66:874–80. doi: 10.1016/j.eururo.2014.01.035, PMID: 24560330 PMC4396847

[25] Iyevleva AG Novik AV Moiseyenko VM Imyanitov EN. EGFR mutation in kidney carcinoma confers sensitivity to gefitinib treatment: a case report. Urol Oncol. (2009) 27:548–50. doi: 10.1016/j.urolonc.2008.03.022, PMID: 18625569

[26] Gross-Goupil M Bernhard JC Ravaud A. Lapatinib and renal cell carcinoma. Expert Opin Investig Drugs. (2012) 21:1727–32. doi: 10.1517/13543784.2012.713935, PMID: 22876762

[27] Warburg O. On the origin of cancer cells. Sci (New York NY). (1956) 123:309–14. doi: 10.1126/science.123.3191.309, PMID: 13298683

[28] Warburg O. On respiratory impairment in cancer cells. Sci (New York NY). (1956) 124:269–70. doi: 10.1126/science.124.3215.269 13351639

[29] Paul S Ghosh S Kumar S. Tumor glycolysis, an essential sweet tooth of tumor cells. Semin Cancer Biol. (2022) 86:1216–30. doi: 10.1016/j.semcancer.2022.09.007, PMID: 36330953

[30] Han HS Kang G Kim JS Choi BH Koo SH. Regulation of glucose metabolism from a liver-centric perspective. Exp Mol Med. (2016) 48:e218. doi: 10.1038/emm.2015.122, PMID: 26964834 PMC4892876

[31] Somsák L. Inhibition of glycogenolysis towards antidiabetic and other therapies. Mini Rev Med Chem. (2010) 10:1091–2. doi: 10.2174/1389557511009011091, PMID: 20716057

[32] Gerich JE. Role of the kidney in normal glucose homeostasis and in the hyperglycaemia of diabetes mellitus: therapeutic implications. Diabetic Med: J Br Diabetic Assoc. (2010) 27:136–42. doi: 10.1111/j.1464-5491.2009.02894.x, PMID: 20546255 PMC4232006

[33] Qi X Li Q Che X Wang Q Wu G. The uniqueness of clear cell renal cell carcinoma: summary of the process and abnormality of glucose metabolism and lipid metabolism in ccRCC. Front Oncol. (2021) 11:727778. doi: 10.3389/fonc.2021.727778, PMID: 34604067 PMC8479096

[34] Huang YQ Liang CH He L Tian J Liang CS Chen X. Development and validation of a radiomics nomogram for preoperative prediction of lymph node metastasis in colorectal cancer. J Clin Oncol: Off J Am Soc Clin Oncol. (2016) 34:2157–64. doi: 10.1200/JCO.2015.65.9128, PMID: 27138577

[35] Sekhar CR Minal Madhu E. Mode choice analysis using random forrest decision trees. Transp Res Procedia. (2016) 17:644–52. doi: 10.1016/j.trpro.2016.11.119

[36] Ucche S Hayakawa Y. Immunological aspects of cancer cell metabolism. Int J Mol Sci. (2024) 25:5288. doi: 10.3390/ijms25105288, PMID: 38791327 PMC11120853

[37] Guo N Luo Q Zheng Q Yang S Zhang S. Current status and progress of research on the ADP-dependent glucokinase gene. Front Oncol. (2024) 14:1358904. doi: 10.3389/fonc.2024.1358904, PMID: 38590647 PMC10999526

[38] Richter S Richter JP Mehta SY Gribble AM Sutherland-Smith AJ Stowell KM. Expression and role in glycolysis of human ADP-dependent glucokinase. Mol Cell Biochem. (2012) 364:131–45. doi: 10.1007/s11010-011-1212-8, PMID: 22219026

[39] Xu H Li YF Yi XY Zheng XN Yang Y Wang Y. ADP-dependent glucokinase controls metabolic fitness in prostate cancer progression. Mil Med Res. (2023) 10:64. doi: 10.1186/s40779-023-00500-9, PMID: 38082365 PMC10714548

