# Supplementary material for: Machine learning-based analysis identifies glucose metabolism-related genes ADPGK as potential diagnostic biomarkers for clear cell renal cell carcinoma
Source: Front Oncol. 2025 Sep 16;15:1559887. doi: 10.3389/fonc.2025.1559887 (PMC12479329; doi:10.3389/fonc.2025.1559887)
Supplement: Supplementary Figure S1 — Colony formation assay. [file DataSheet1.docx]

Supplementary material


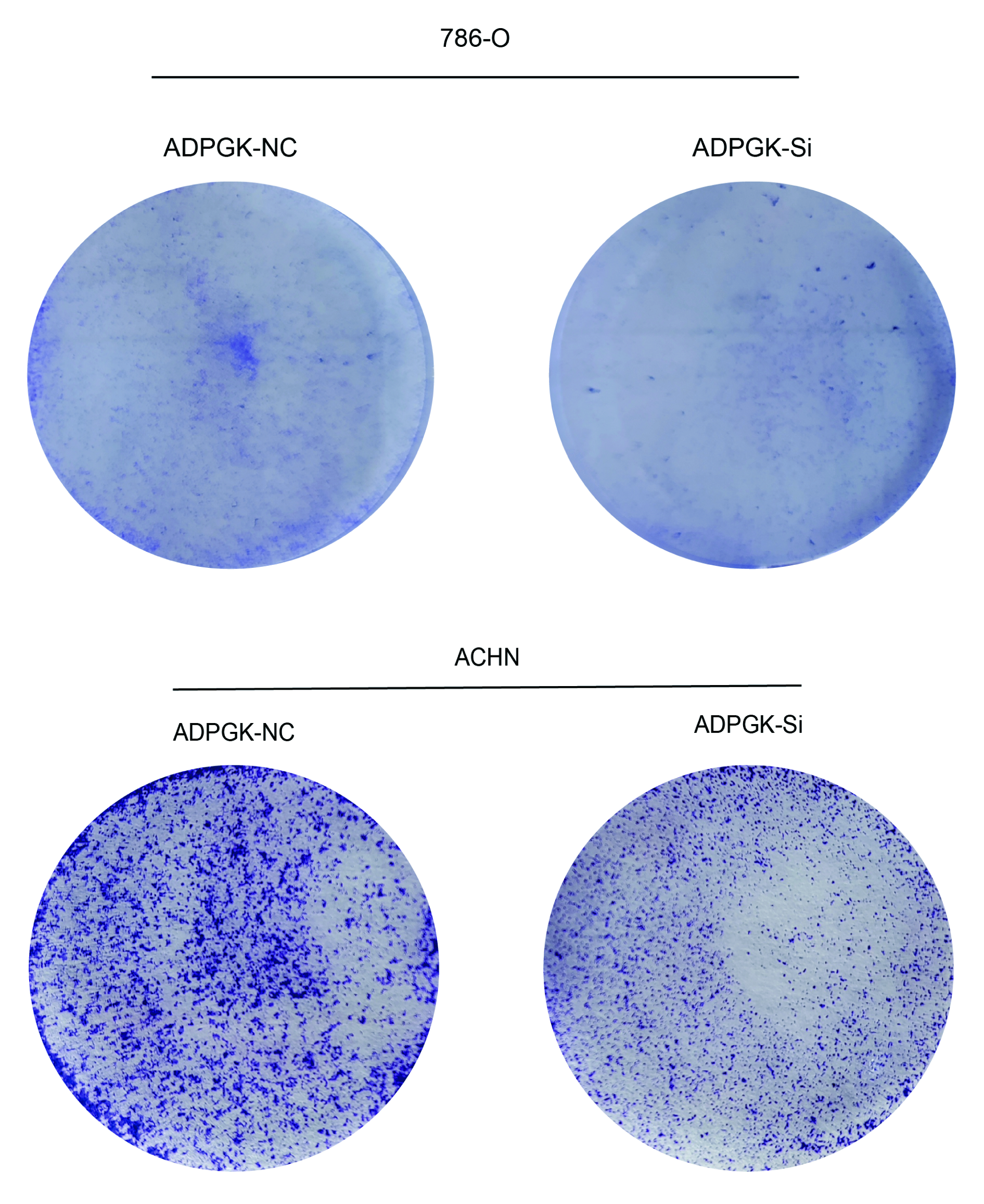


Figure S1

Colony formation assay showed that down-regulation of ADPGK inhibited the proliferative activity of 786-O and ACHN cells as compared to the si-NC group.


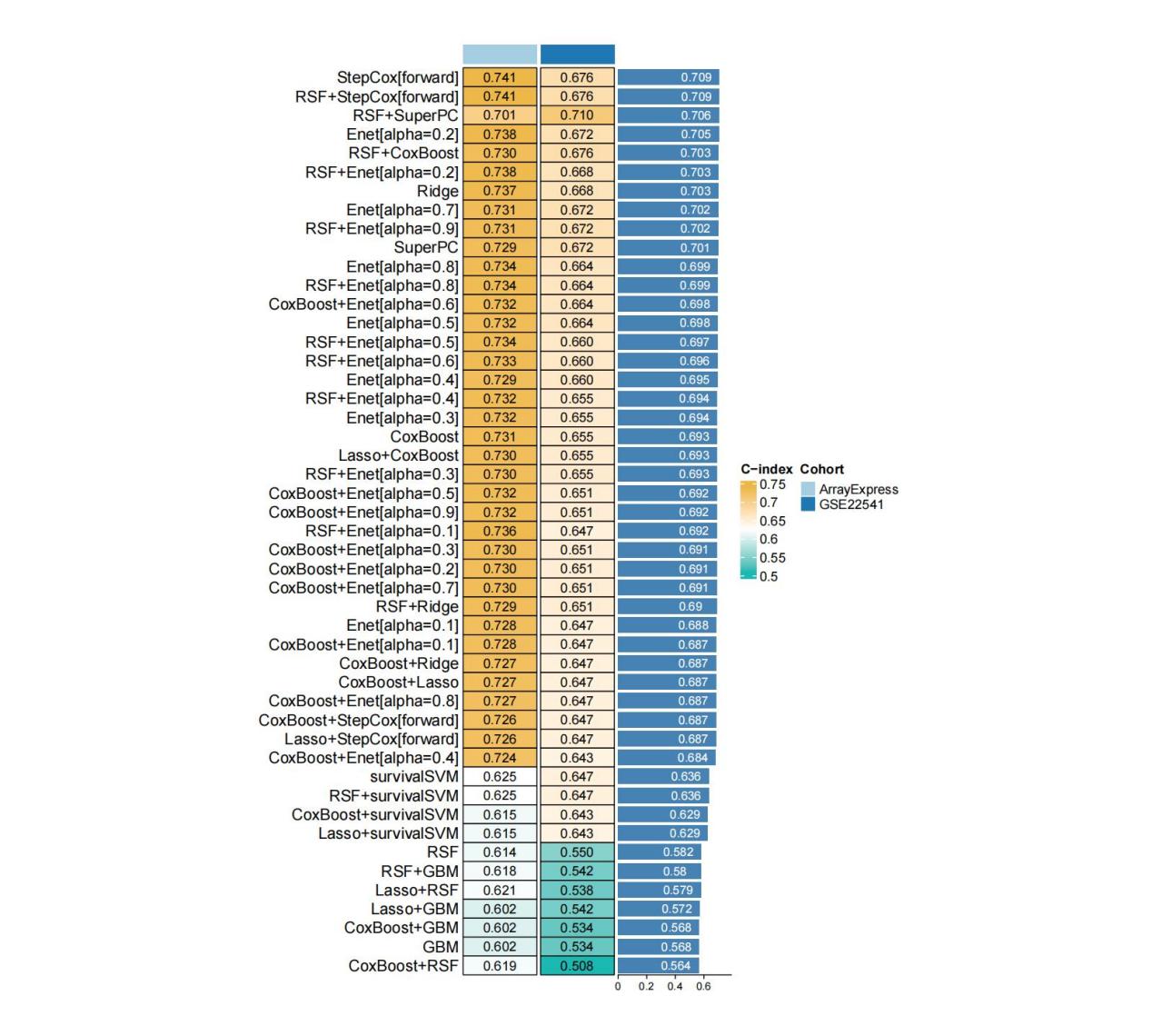


Figure S2

Machine learning results indicate that among the 7 screened genes—AAAS, ADPGK, FBP1, HK3, NUP85, NUP93, and SLC25A13—the combination of RSF and stepCOX machine learning models demonstrated the best prognostic prediction capability for patients. The predicted C-index value reached 0.741 in the ArrayExpress dataset and 0.676 in the GEO database, suggesting excellent predictive performance. Additionally, validation results from external datasets were successfully completed.

A B


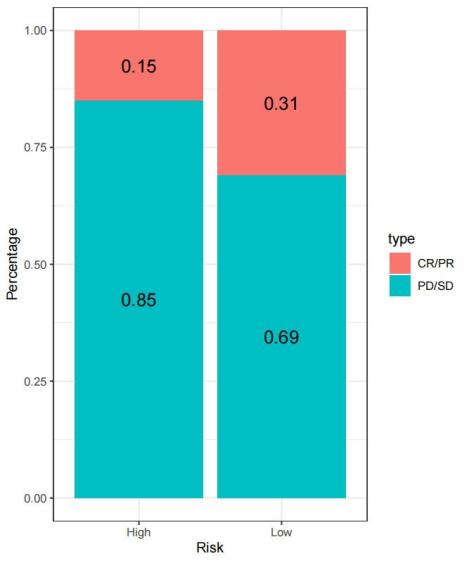

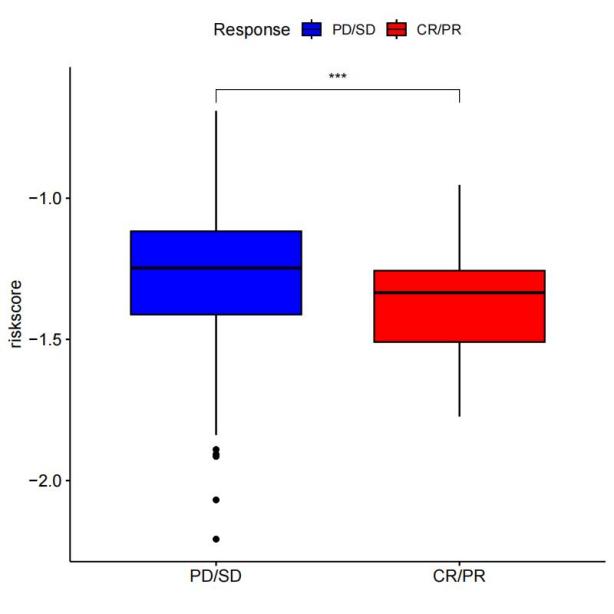


Figure S3(A-B)

Based on the IMvigor210 dataset[1], we stratified the risk scores derived from machine learning algorithms into high and low groups, and compared the proportions of immunotherapy outcomes between these two groups. The results indicate that the low-risk score group exhibited a higher overall proportion of CR/PR cases, while the CR/PR subgroup demonstrated lower risk score levels. This suggests that lower risk scores may correlate with better immunotherapy efficacy.


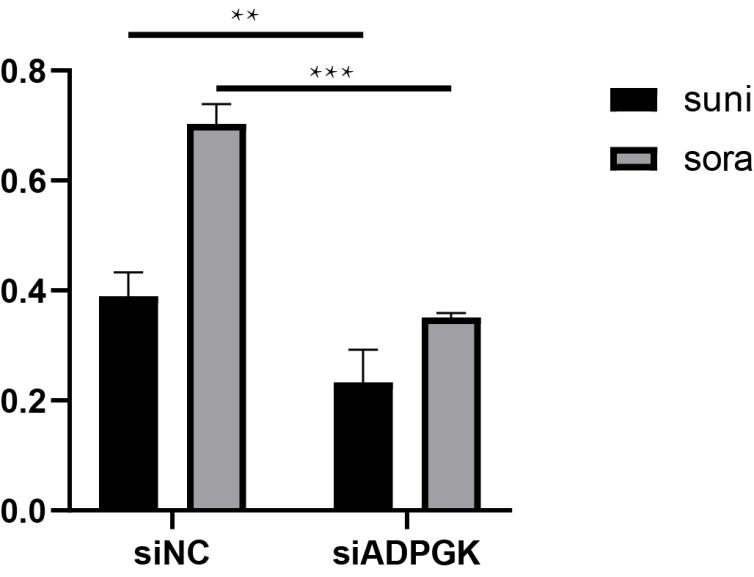


Figure S4

After siRNA treatment of 786-O cells, we administered sorafenib (1mM) and sunitinib (1mM), followed by CCK8 assay after 24 hours to calculate cell viability. We observed that both sunitinib and sorafenib exhibited higher drug sensitivity after siADPGK knockdown.


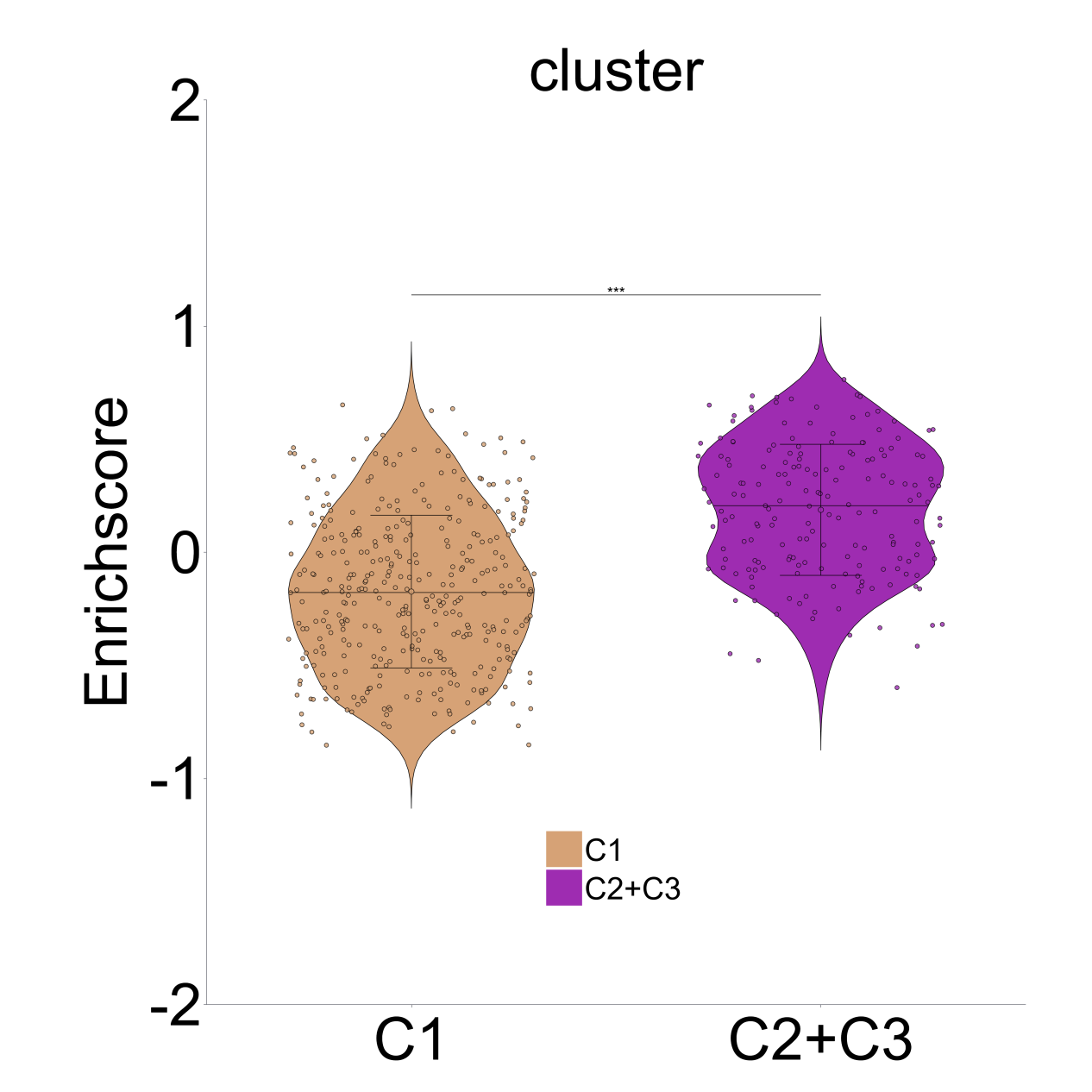


Figure S5

After merging clusters C2 and C3, their enrichment scores were compared with those of C1. The violin plot illustrates the comparison results. Considering there is a significant difference between the two groups, Parazacco spilurus subsp. spilurus, merging C2 and C3 into one group shows no obvious issues. The manuscript subsequently uniformly refers to C2+C3 as C2.

Data access

All code applied in the paper has been uploaded to Github(https://github.com/Booooom21/ADPGK), with some analysis results completed on the Sangerbox platform[2].

Reference

[1] Rosenberg JE, Galsky MD, Powles T, Petrylak DP, Bellmunt J, Loriot Y, Necchi A, Hoffman-Censits J, Perez-Gracia JL, van der Heijden MS, Dreicer R, Durán I, Castellano D, Drakaki A, Retz M, Sridhar SS, Grivas P, Yu EY, O'Donnell PH, Burris HA, Mariathasan S, Shi Y, Goluboff E, Bajorin D. Atezolizumab monotherapy for metastatic urothelial carcinoma: final analysis from the phase II IMvigor210 trial. ESMO Open. 2024 Dec;9(12):103972. doi: 10.1016/j.esmoop.2024.103972

[2] Chen D, Xu L, Xing H, Shen W, Song Z, Li H, Zhu X, Li X, Wu L, Jiao H, Li S, Yan J, He Y, Yan D. Sangerbox 2: Enhanced functionalities and update for a comprehensive clinical bioinformatics data analysis platform. Imeta. 2024 Sep 2;3(5):e238. doi: 10.1002/imt2.238. PMID: 39429873; PMCID: PMC11487553.
